# Microalgae *Chlorella vulgaris* biomass harvesting by natural flocculant: effects on biomass sedimentation, spent medium recycling and lipid extraction

**DOI:** 10.1186/s13068-018-1183-z

**Published:** 2018-06-28

**Authors:** Liandong Zhu, Zhaohua Li, Erkki Hiltunen

**Affiliations:** 1 School of Resource and Environmental Sciences, Wuhan University, 129 Luoyu Road, Wuhan, 430079 People’s Republic of China; 2 Faculty of Technology, University of Vaasa and Vaasa Energy Institute, P.O. Box 700, FI-65101 Vaasa, Finland; 3 Hubei Collaborative Innovation Center for Green Transformation of Bio-Resources, Faculty of Resources and Environmental Science, Hubei University, Wuhan, 430062 People’s Republic of China

**Keywords:** *Chlorella vulgaris*, Natural flocculant, Biomass harvesting, Coagulation and flocculation, Lipid extraction, Chitosan

## Abstract

**Background:**

Microalgal biomass harvesting using traditional chemicals is costly for the production of biofuels, hindering the scale-up process of the technology. Thus, the search for a cost-effective microalgal harvesting method is extremely important. Using chitosan as a natural flocculant to harvest microalgal biomass seems to be an efficient and convenient solution. Although microalgal biomass flocculation by chitosan has been reported in some previous studies, literature on the harvesting of microalgae *C. vulgaris* biomass using such polymer is scanty. In addition, there is limited information available on whether the usage of chitosan during the harvesting will affect downstream lipid extraction. Still, whether microalgae can be re-grown with the spent medium after chitosan flocculation is still unknown.

**Results:**

In this study, microalgal biomass harvesting using chitosan as a natural flocculant and aluminum sulfate as a traditional flocculant was compared and evaluated. Optimal doses and effects on biomass sedimentation, spent medium recycling and lipid extraction were investigated. The results showed that the optimal doses for chitosan and aluminum sulfate to achieve more than 90% biomass recovery were 0.25 and 2.5 g/L, respectively. The sedimentation time of 10 min was found to be the most appropriate to remove over 90% biomass from culture. The spent medium after chitosan flocculation could be potentially recycled for the re-cultivation of microalgae, which demonstrated robust growth in comparison with those grown in the recycled medium from aluminum sulfate flocculation. The lipid content of microalgae harvested by chitosan reached 32.9, 4.6% higher than that of those harvested by aluminum sulfate, indicating that the application of the natural flocculant would not impact the downstream extraction of microalgal lipids.

**Conclusion:**

The results herein presented, demonstrated that chitosan is applicable for microalgal harvesting during the upscaling process. Flocculation method developed by using chitosan as a natural flocculant is a worthy microalgal harvesting method for microalgae-based biofuel production. There is hope that chitosan can be reasonably and technically realistically applied in a full-scale process for the harvesting of microalgal biomass.

## Background

Today almost 80% of global energy is derived from fossil sources [1], and thus energy shortage together with global warming and climate changes has triggered the search for renewable and sustainable energy sources [2–5]. Of the various alternative sources, microalgae have received a lot of attention as a biofuel feedstock. Microalgae utilize water, CO_2_ and sunlight to produce biomass that can be harnessed for the production of products from food to fuels [6, 7]. Microalgae grow very fast, almost 20 times faster in comparison with oily plants such as rapeseed and corn, which typically contain no more than 5% oil of the total biomass. In contrast, the lipid contents of most microalgal species have been known to be 20–40% of dried weight, and some species might contain the lipids up to 60% or even more under some specific cultivation conditions [8]. Other advantages of microalgae as a biofuel feedstock lie in the noncompetition with food for farmland, ability to be grown with wastewater and waste CO_2_, and so forth [9, 10].

Microalgal cells are negatively charged and very tiny with the size range between 5 and 50 μm [11]. Microalgal cells are easily suspended in the culturing medium, since their negative charges prevent aggregation. Therefore, the removal of such small cells from the culture is highly energy intensive and costly for the production of microalgal biofuels [12–14]. The common methods for microalgal biomass harvesting include centrifugation, filtration, flotation and flocculation.

Of those techniques, flocculation is considered as an effective, convenient and preferable process for the harvesting of microalgal biomass [15]. During flocculation, cationic flocculants with positive charges neutralize the negative charges of microalgal cells, forming aggregates or larger particles. Afterwards, the aggregates coalesce into large flocs, which settle via gravitational forces. The flocculation efficiency is subject to the nature of flocculants, pH and salinity of the medium and the species of microalgae and their charge densities, cell concentrations, growth phase, and so forth [1, 16]. A large number of chemicals have been applied as flocculants (Table 1), which include various inorganic metal salts such as ferric chloride, aluminum sulfate and ferric sulfate [17, 18], and organic polymers such as cationic starch [19] and polydiallyldimethylammonium chloride [20]. In addition, the choice of flocculant also depends on the targeted products of microalgal biomass. Inorganic metal salts has the limitation in their usage, since they might contaminate the microalgal biomass together with effluent quality during flocculation. The application of some chemical flocculants might also adversely affect the biochemical components such as proteins, starch and lipids [21], leading to unfeasibility for food or nutrition production. To overcome these issues, natural polymers or natural flocculants such as chitosan, *Moringa oleifera* seed flour and guar gum have recently been applied for the harvesting of microalgal biomass, and some promising results have been obtained [22–24].

**Table 1:** Comparison of inorganic and organic flocculants for microalgal biomass harvesting [25]

Parameters	Inorganic flocculants	Organic flocculants
Nature of flocculants	Multivalent salts	Polyelectrolytes/polymers
Key characteristics of an effective flocculant	Increasing molecular weight can increase the binding capabilities	Flocculants that have a high charge density are therefore more effective
Sensitivity to pH	Coagulation using inorganic coagulants is highly sensitive to pH level	Coagulation using organic coagulants is less sensitive to pH
Sensitivity to biomass concentration	Highly sensitive to concentration	Highly sensitive to concentration
Dosage of flocculants required	A large concentration of inorganic flocculant is needed in order to maintain flocculation efficiency, and may contaminate the end product (e.g., addition of aluminum and iron salts)	Lower dosages of organic flocculants are required, and less or no contamination occurs
Applicability	Although some coagulants may work for some microalgal species, they do not work for others	Wide range of applications for larger number of microalgal species

In general, chitosan is a cationic polyelectrolyte derived by the deacetylation of chitin. Traditionally, chitosan has been suggested as a natural flocculant for wastewater treatment, since it is non-toxic, non-corrosive, biodegradable, safe to handle and has attractive adsorption and flocculation ability [22, 26, 27]. Due to high cationic charge density, chitosan can strongly absorb the negatively charged microalgal cells onto its surface, and this mechanism might lie in polymer bridging and/or charge neutralization. Xu et al. [28] investigated the chitosan flocculation of the green microalga *Chlorella sorokiniana*, and suggested that the relative clarification efficiency could reach above 99% below the pH value of 7. Dewatering of the green microalgae *Neochloris oleoabundans* through chitosan flocculation was investigated by Beach et al. [29], who obtained an optimum dose of 100 mg/L. In another study, it was found that the structural modification of chitosan by grafting or inserting the copolymers could improve the harvesting efficiency, due to the increase of positive charge and molecular weight [30]. Although the application of chitosan as a natural flocculant has been reported in some previous studies, literature on the harvesting of microalgae *C. vulgaris* biomass using such polymer is scanty. In addition, there is limited information available on whether the usage of chitosan during harvesting will affect downstream lipid extraction. This is important because apart from biomass recovery improvement, flocculants are not supposed to hinder downstream bioenergy production. Still, the flocculation by the application of chitosan might affect the final effluent quality, and whether microalgae can be re-grown with the spent medium after chitosan flocculation is still unknown.

In this study, the performance of chitosan as a natural flocculant to harvest *C. vulgaris* biomass was evaluated and compared with the inorganic flocculant aluminum sulfate. Effects of chitosan and aluminum sulfate application on biomass sedimentation, spent medium recycling and lipid extraction were accordingly investigated. The objectives of this study were: (1) to evaluate chitosan flocculation performance in the harvesting of *C. vulgaris* biomass and determine its optimal dose, (2) to disclose the sedimentation properties of *C. vulgaris* biomass flocculated by chitosan, (3) to reveal the recyclability of the spent medium from flocculation to regrow microalgae and (4) to assess the potential influences of flocculant application on lipid extraction during downstream process. In addition, the feasibility of using chitosan as a natural flocculant in a large-scale process was also discussed.

## Results and discussion

### Optimal dose of natural flocculant

**Fig. 1:**
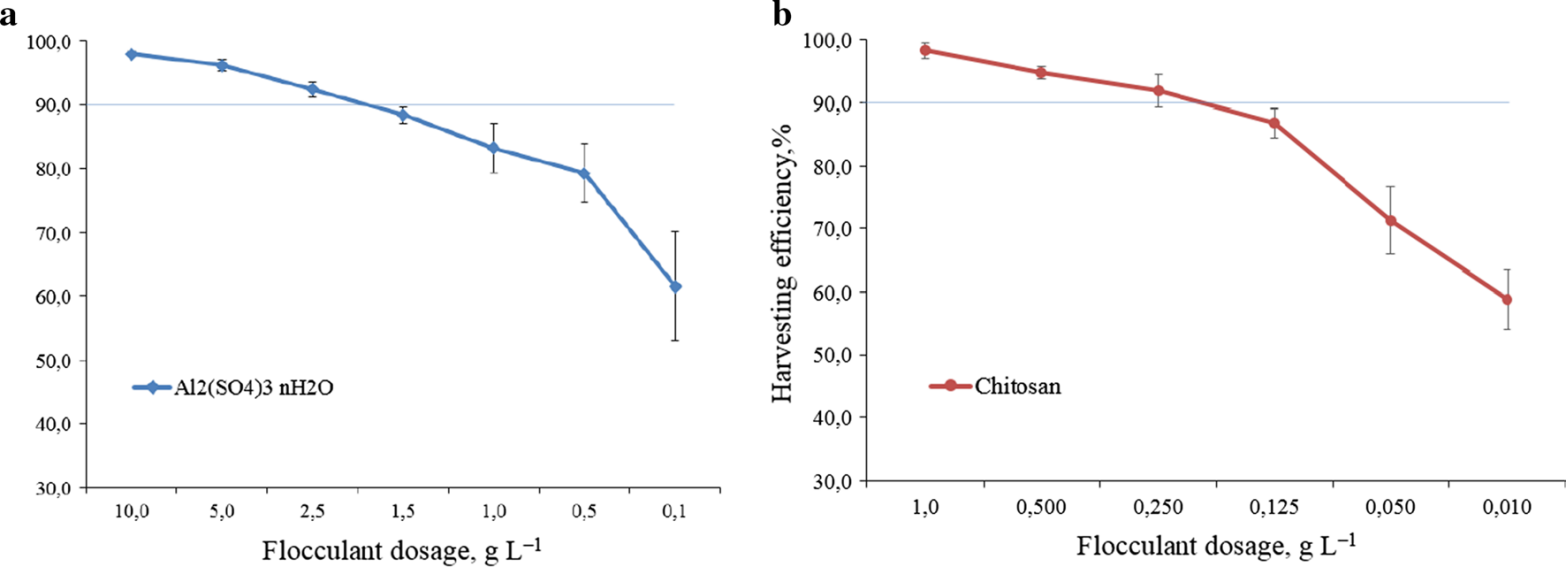
Microalgae *Chlorella vulgaris* biomass harvesting using chitosan as a flocculant in comparison with aluminum sulfate under different dosages. **a** aluminum sulfate; **b** chitosan

In an attempt to enhance microalgal biomass recovery, the coagulation–flocculation process was applied using chitosan. Since biomass concentration is a factor that affects flocculation efficiency [17], identical microalgal suspension with the same biomass concentration was initially applied in this part of investigation. It was found that microalgal biomass harvesting efficiency for aluminum sulfate and chitosan ranged from 61.5 to 98.0% and from 58.8 to 98.3%, respectively (Fig. 1). As the increase of flocculant dose, the biomass recovery efficiency increased. In comparison with the tested conventional chemical flocculant, around 10-times lower dosage was needed for chitosan as the natural flocculant, thanks to the chemical nature of the compound. Chitosan has a high cationic charge density, and thus can strongly absorb the negatively charged microalgal cells onto its surface through polymer bridging and charge neutralization. de Godos et al. [31] compared microalgal biomass harvesting efficiency between ferric metal salts and organic polymers, and found that 5- to 6-times lower dosages were required for organic polymers. In another study, much lower doses were achieved when polydiallyldimethylammonium chloride was used to harvest *Chlorella vulgaris* and *Nannochloropsis salina* [20]. However, comparison is not straight forward, since harvesting efficiency strictly depends on the concentration of microalgal biomass.

In this study, the lowest concentration of flocculant ensuring over 90% biomass recovery was recognized as the optimal dosage. Regarding aluminum sulfate, 92.4% of microalgal biomass was recovered with a dosage of 2.5 g/L. In contrast, a much lower dose of chitosan (0.25 g/L) obtained a similar efficiency of 91.9%. In other words, chitosan could replace conventional chemical flocculant to obtain an enhanced biomass recovery (Table 2). Similar to this study, Kothari et al. [32] used egg shell to prepare natural flocculant and obtained the optimized natural flocculant dosage of 100 mg/L, which could remove more than 98% biomass from microalgal solution during 30 min. Gutiérrez et al. [33] applied natural cationic flocculants (ecotan and tanfloc) extracted from the bark of *Acacia mearnsii*, and suggested that low flocculant doses of 10 and 50 mg/L of ecotan and tanfloc enabled over 90% biomass recovery, respectively.

**Table 2:** Efficiency comparison of several common flocculants for microalgal biomass harvesting

Flocculants	Optimal dosage, g/L	Species	Algal biomass concentration, g/L	Efficiency, %	References
Chitosan	0.25	*Chlorella vulgaris*	1.2	91.9	This study
Chitosan	0.05	*Chlorella vulgaris*	0.5	92.3	This study
Cationic inulin	0.06	*Botryococcus* sp.	–	88.6	[34]
Cationic cassia gum	0.08	*Chlamydomonas* sp.	0.85	92	[24]
Cationic starch	0.03	*Scenedesmus dimorphus*	0.12	91	[19]
Aluminum sulfate	2.5	*Chlorella vulgaris*	1.2	92.4	This study
Aluminum sulfate	0.8	*Scenedesmus spinosus*	0.4	99.1	[18]
Ferric sulfate	1.5	*Scenedesmus spinosus*	0.4	61.6	[18]
Ferric chloride	1.1	*Chlorococcum* sp.	–	92	[21]
Iron oxide	0.3	*Chlorella ellipsoidea*	0.8	90	[35]
Yttrium iron oxide	2.5	*Chlorella vulgaris*	0.82	93	[36]

The microalgal biomass concentration used during the experiments is a critical parameter. In this respect, the effect of microalgal cell concentration on biomass recovery needs to be investigated. In order to form sample suspensions with various cell concentrations, microalgal cells were harvested through centrifugation at 3000 rpm, and subsequently re-suspended in the fresh medium. Afterwards, flocculation experiments of these suspensions were conducted. Table 3 shows the relationship between cell concentration and chitosan dosage during the microalgal biomass harvesting. It can be seen that the optimal chitosan dose for over 90% biomass recovery increased almost proportionally from 0.050 mg/L to 0.250 g/L as the increase of microalgal biomass concentration from 0.5 to 1.2 g/L. Therefore, cell concentration is widely recognized as a critical parameter to determine the optimal chitosan dosage [28], since all parameters except microalgae concentration were identical in the sample suspensions.

**Table 3:** Relationship between cell concentration (1.2, 0.8 and 0.5 g/L) and chitosan dosage during microalgal biomass harvesting (mean ± SD)

Biomass concentration in dried weight, g/L	Dose, g/L	Harvest efficiency, %
1.2	1.000	98.3 ± 1.3
	0.500	94.7 ± 1.0
	0.250	91.9 ± 2.6
	0.125	86.8 ± 2.4
	0.050	71.3 ± 5.4
	0.010	58.8 ± 4.6
0.8	1.000	98.0 ± 0.9
	0.500	97.5 ± 2.3
	0.250	98.7 ± 1.2
	0.125	91.2 ± 0.9
	0.050	80.8 ± 2.6
	0.010	65.4 ± 5.0
0.5	1.000	98.9 ± 0.4
	0.500	97.5 ± 1.9
	0.250	96.3 ± 0.5
	0.125	94.6 ± 1.6
	0.050	92.3 ± 2.5
	0.010	78.9 ± 4.3

### Sedimentation of microalgal biomass

**Fig. 2:**
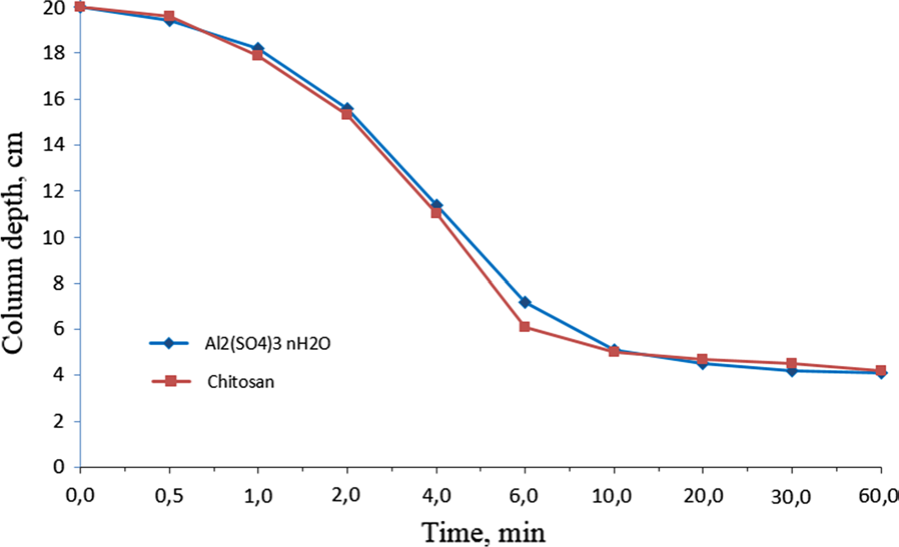
The changes of column depths at different time intervals during settling by employing chitosan as a flocculant in comparison with aluminum sulfate

Sedimentation is a quiescent process that allows the formed flocs to settle under the influence of gravity. In this part of investigation, settling tests were conducted after the flocculation of microalgal biomass using chitosan and aluminum sulfate. The changes of column depths at different time intervals during settling are exhibited in Fig. 2.

As shown in Fig. 2, there was no evident difference in the settling of flocculated biomass between treatments by natural flocculant and traditional flocculant. The column depth of the formed flocs reduced gradually in the first minute, after which a rapid decrease occurred until 6th min. During the period between the 1st and 6th min, the relative settling velocity for aluminum sulfate and chitosan was identically 0.4 mm/s. On contrast, without any flocculant addition the velocity obtained along the settling column was fairly constant and only 0.008 mm/s (data not shown in figure), which was much lower than those achieved in this study. The findings in this study were in line with the results by Gutiérrez et al. [33], who applied natural flocculants (ecotan and tanfloc) to harvest microalgal biomass from wastewater treatment systems and obtained the according velocities of 0.21–0.56 mm/s and 0.16–0.35 mm/s for ecotan and tanfloc, respectively. As from 6 min, the settling speed slowed down and reached a relatively stationary level after 10 min. The determination of the optical densities of supernatants indicated more than 90% of the biomass recovery for aluminum sulfate or chitosan flocculation during the sedimentation within 10 min, which was present as the optimal sedimentation time. Sirin et al. [37] applied aluminium sulphate and poly-aluminium chloride to harvest *Nannochloropsis gaditana* biomass, and suggested that after 15 min of settling time no more settling column addition was found.

### Recycling spent media to re-grow microalgae

**Fig. 3:**
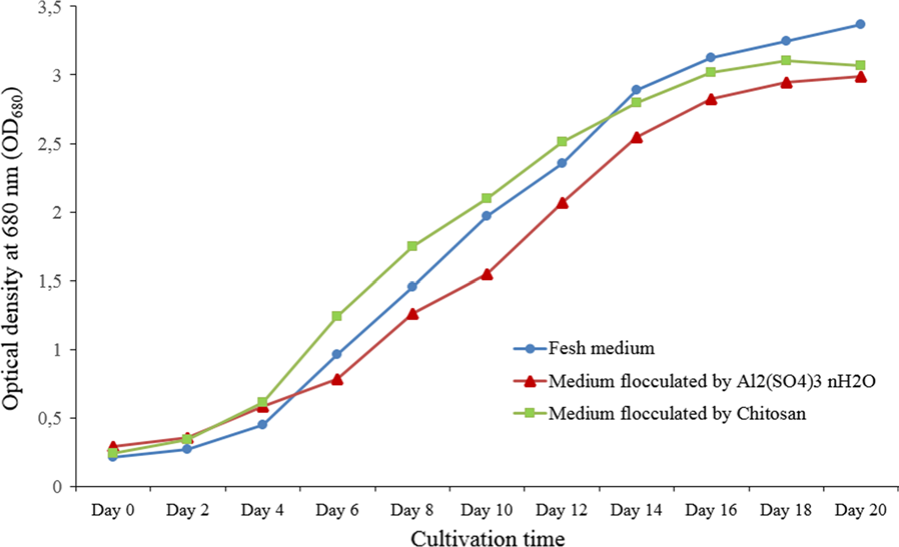
Comparison of microalgae *Chlorella vulgaris* grown in recycled and fresh media

Harvesting water recycling from microalgal production system to re-grow microalgae could not only save water resource but also recover nutrients that left during the harvesting process [38]. In this part of investigation, the media were recovered after flocculation by chitosan and aluminum sulfate, and then a certain amount of nutrient components from the modified Bristol medium was added for the purpose of supplementation. The spent media together with the fresh medium were used to re-cultivate *C. vulgaris*. The characteristic comparison of microalgae grown in recycled and fresh media is shown in Fig. 3.

As shown in Fig. 3, microalgae grown in the spent media from both chitosan and aluminum sulfate flocculation demonstrated robust growth. However, in comparison with the sulfate-flocculated medium, microalgae in the chitosan-flocculated medium experienced better growth in biomass accumulation, especially during the cultivation period between day 6 and 12. The optical density of microalgae *C. vulgaris* grown in the recycled media from chitosan flocculation was very close to that grown in fresh medium, indicating that the spent medium after chitosan flocculation could be potentially recycled for the re-cultivation of microalgae. Previous studies also concluded that harvesting water could be recycled to re-grow microalgae such as *Scenedesmus* sp., *Chlorella zofingiensis* and *Chlorococcum* sp., when either centrifugation or flocculation was applied as the method [21, 39, 40]. In addition, the lag phase of microalgal growth was shortened in the treatments with spent media, since the recycled medium still contained some un-harvested microalgal cells, possibly accelerating the growth of microalgae [40]. Another reason might lie in the fact that the un-harvested microalgal cells had already adapted to the medium environment, facilitating microalgal cells to utilize nutrients available.

### Effects of natural flocculant application on lipid extraction

**Fig. 4:**
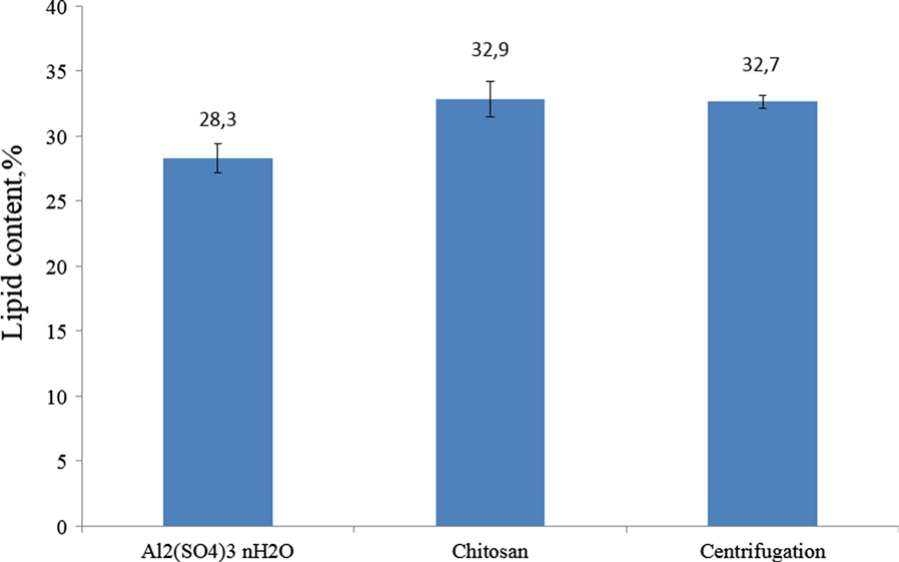
The effects of centrifugation and flocculation fulfilled by chitosan and aluminum sulfate on the extraction of lipids

The addition of chemicals into microalgal suspension to induce biomass flocculation might modify biomass characteristics and cause biomass contamination, probably influencing the downstream processes such as anaerobic digestion [33]. To the best of our knowledge, in literature there is limited information available on the effect of natural flocculant application on microalgal lipid extraction. In this part of investigation, the comparison of the effects of using centrifugation and natural flocculant as the harvesting methods on the extraction of microalgal lipids was conducted, and the results are shown in Fig. 4.

As shown in Fig. 4, the utilization of chitosan as the flocculant would not affect the extraction of microalgal lipids, the content of which reached 32.9%. In contrast, the application of aluminum sulfate as the flocculant to harvest *Chlorella* biomass resulted in 4.4% reduction of lipid contents, compared with centrifugation as the method. This is because some portions of aluminum sulfate might be attached onto microalgal cells and settle with the flocs formed. The remained substances in harvested microalgal biomass affected the purity and thus the content of lipids extracted. Another reason might result from the impact of toxicity or interference of residual metal in the harvested biomass during the lipid extraction. Similar to the present study, Choi [41] found that egg shell solution has non-toxic effect on microalgal cells during the flocculation process. No disintegration was found in cell surface of *C. vulgaris* biomass flocculated by poly glutamic acid under room temperature conditions [42]. According to Ummalyma et al. [21], the fatty acids profile of the biomass showed differences when using ferric chloride and aluminum sulfate to flocculate microalgal cultures, while there was no effect when biomass was auto-flocculated. In another study, Balasubramanian et al. [43] suggested that transition metal ions such as Fe and Cu were effective catalysts for the free radical oxidation of lipids, likely causing the loss of a certain amount of lipids in this study. However, using chitosan as the natural flocculant would not demonstrate any negative effects, which presented as an added advantage.

### Feasibility of using natural flocculant in a large-scale process

To achieve more than 90% biomass recovery, the previous sections show that the residence time (T) for flocculation-sedimentation process is 26 min (0.018 day) in this study (1 min aggregation, 15 min flocculation and 10 min sedimentation). In an effort to promote the natural flocculant application in a large-scale biomass harvesting process, the technical feasibility of such a system needs to be assessed in terms of the required volume or capacity [44].

In this study, we assume that raceway ponds serve as the large facility and the total pond surface area (*A*) of raceways for the production of microalgal biomass is 10,000 m^2^ (1 ha). According to Chisti [45], it is realistic for such a facility to achieve the dry biomass productivity (*P*) of 0.020 kg/m^2^/day and it is typical to obtain the dry biomass concentration of 0.5 kg/m^3^. Therefore, the volumetric production rate (Vr) of microalgal biomass for such a facility can be estimated using the following equation:

$${\text{Vr}} = AP/C = 1000 \times 0.020/0.5 = 400\;{\text{m}}^{ 3} / {\text{day}}$$

To process this volume of biomass through a chitosan-based flocculation–sedimentation system, the required volume of the system (Vs) can be calculated using the following formulae:

$${\text{Vs}} = {\text{VrT}} = 400 \times 0.018\,\, = 7.2\;{\text{m}}^{ 3}$$

According to Chatsungnoen and Chisti [46], the culture depth of raceways was typically 0.25 m, and thus the total working volume of raceways will be 2500 m^3^ (10,000 × 0.25). Hence, it is worthy of note that the required volume of the flocculation–sedimentation system (Vs) is only 0.3% of the total working volume of raceways, demonstrating that it is reasonable and clearly technically realistic to use chitosan in a large-scale process for the harvesting of microalgal biomass. It is extremely possible to achieve the annual biomass production of nearly 60 tons (= 0.5 × 400 × 0.90 × 365 × 0.90 × 10^−3^) if the system is operated for 90% of days throughout a year. Although this part of investigation emphasizes the high potential of chitosan as a natural flocculant for biomass recovery, the validation under large-scale conditions is still required in practice. Upon large-scale validation, the adjustment of an optimal dosage might be required. However, dosage lower than 50 mg/L might be sufficient in a large-scale process, since chitosan dose of 50 mg/L was achieved to harvest 0.5 g/L biomass in this study.

In this study, the optimal dose of chitosan to harvest 0.5 g/L biomass was found to be 50 mg/L, which, in other words, indicated 100 g/kg of dry weight biomass for the efficiency. Assuming that the current chitosan price is still 7 US$/kg [47], the cost for *C. vulgaris* biomass harvesting will be 0.7 US$/kg of dry weight biomass, which shows limited economic advantage in the comparison of metal salts application as the flocculants. However, chitosan is environmentally friendly, efficient and non-toxic for the harvesting of microalgae, and thus using chitosan as a flocculant will be a competitive and suitable method in future if the costs can be shortened with the help of research and technology advancement. Harvesting efficiency improvement together with chitosan production cost reduction through technology development and process optimization will have the main roles to play, in an effort to promote the economics of microalgal biomass flocculation using chitosan. It is only a matter of time, and eventually microalgal harvesting with chitosan will become economically convenient in future.

## Conclusions

To achieve more than 90% of microalgae *C. vulgaris* biomass recovery for the harvesting of the biomass with the concentration of 1.2 g/L, the optimal dosage of chitosan as a natural flocculant was 0.25 g/L, which was 10-times lower than that for aluminum sulfate. During the sedimentation, the settling velocity of the period between the 1st and 6th min for aluminum sulfate and chitosan identically reached 0.4 mm/s. The appropriate time for microalgal biomass to settle was 10 min, which allowed more than 90% of the biomass recovery. Microalgae grown in the spent medium from chitosan flocculation demonstrated robust growth, and its optical density throughout the growth phase was very close to those grown in fresh medium, indicating that the spent medium could be potentially recycled for the re-cultivation of microalgae. The utilization of chitosan as the natural flocculant would not affect the downstream extraction of microalgal lipids, while aluminum sulfate would, leading to 4.4% reduction of lipid contents. The feasibility discussion showed that it is reasonable and clearly technically realistic to use chitosan in a large-scale process for the harvesting of microalgal biomass. To promote chitosan as an ideal material for microalgal biomass harvesting during the large-scale production, further research is needed to underpin its technical feasibility through the investigation of many experimental parameters (e.g., agitation speed, pH adjustment and nitrogen concentration of the medium during cultivation) that affect the harvesting efficiency. In addition, the economic viability evaluation of the use of such flocculant for bulk microalgal harvesting is also required in the future research.

## Methods

### Microalgal biomass production

Oleaginous microalgae *C. vulgaris* was obtained from the biological lab of the Tampere University of Technology in Finland and preserved in N8 medium. After inoculation, the species was cultivated in autoclaved modified Bristol medium, containing NaNO_3_ (2.94 mmol/L), CaCl_2_·2H_2_O (0.17 mmol/L), MgSO_4_·7H_2_O (0.30 mmol/L), K_2_HPO_4_ (0.43 mmol/L), KH_2_PO_4_ (1.29 mmol/L), NaCl (0.43 mmol/L) and FeSO_4_·2H_2_O (0.01 mmol/L). The pH value of culturing medium was regulated to 6.8. Conical flasks (1L, working volume of 700 mL) were served as photobioreactors to grow microalgae. To provide a carbon source, a certain amount of bicarbonate of 2 g/L was added into the medium. The flasks were laid on an open-air platform shaker (MaxQ 2000, Barnstead, USA) with the rotating speed of around 220 rpm for culture mixing, and the cultivation was conducted in a ventilating chamber in the lab, where the temperature was maintained at around 23 °C. Flasks were continuously illuminated by cool white fluorescent lamps with the light intensity of around 75 μmol/m^2^/s. Microalgae were cultivated for 20 days to achieve the dried biomass of 1.2 g/L, and the pH value of the culture reached around 9.5 in the end.

### Flocculant materials

The natural flocculant applied in this study was dry chitosan powder with medium molecular weight of 190,000–310,000, and it was purchased from Sigma Aldrich (Germany). The chitosan aqueous stock solution (5 g/L) was prepared by dissolving chitosan in 1% acetic acid solution under continuous agitation assisted with a magnetic stirrer at 100 rpm for over 24 h until a clear solution was obtained. As a comparison, a traditional flocculant which was aluminum sulfate (Al_2_(SO_4_)_3_·nH_2_O) was purchased from VWR Co. LLC, USA and applied in this study. Flocculation is normally performed after coagulation of the biomass by neutralizing the charges on their surfaces. Chitosan, which is a polyelectrolyte with high cationic charge density, can strongly absorb the negatively charged microalgal cells onto its surface through charge neutralization and polymer bridging. Therefore, in this study no other coagulant was applied during the microalgal biomass harvesting by chitosan.

### Jar test for the optimal dosage determination

Through the addition of a certain amount of chitosan stock solution, the investigated dosages of chitosan applied for microalgal biomass harvesting were 1.00, 0.50, 0.25, 0.125, 0.05 and 0.01 g/L. As for aluminum sulfate, the dose level applied in this part was 10.0, 5.0, 2.5, 1.5, 1.0, 0.5 and 0.1 g/L. Different dosages of flocculants were added into vials, which contained 20 mL microalgal solution. The mixtures were rotated assisting by magnetic stirrers at the speed of 150 rpm for 1 min for aggregation and then 25 rpm for 15 min for flocculation, after which aggregates were settled for 15 min. Afterwards, the supernatant was sampled for the determination of optical density (OD) at 680 nm using a UV–Visible spectrophotometer (UV–1601, Shimadzu, Japan). The formula applied for the harvesting efficiency (biomass recovery) is shown in Eq. (1):

1 $${\text{Harvesting}}\;{\text{efficiency}} = {\text{H}}\% = 100\% \times \left( {{\text{OD}}_{0} - {\text{OD}}_{\text{i}} } \right)/{\text{OD}}_{0}$$

where OD_0_ and OD_i_ are defined as the mean OD values of the initial culture before harvesting and the supernatant after harvesting, respectively.

### Sedimentation of microalgal biomass

In order to measure the settling property of the formed flocs, static column (height, 20 cm; internal diameter, 2.6 cm) settling experiments were carried out, following the standard methods applied in the field of wastewater treatment [48]. The procedure applied was as follows: first, microalgae were accordingly coagulated and flocculated in vials by adding the flocculants with the optimal doses that had already been achieved in the previous section; second, the formed flocs were gently poured into each column to prevent any breakage; third, column depth of the formed flocs at different time intervals over 1 h (0.5, 1, 2, 4, 6, 10, 20, 30 and 60 min) was immediately measured.

### Recycling of flocculated medium for microalgal re-cultivation

After flocculation and sedimentation, the flocs settled and the cultivation medium were separated. The pH value of the cultivation medium was adjusted to the original level by adding a certain amount of HCl. After microalgal biomass cultivation and harvesting, the nitrogen and phosphate contents of flocculated medium were found to be 0.62 and 0.12 mmol/L, respectively. To keep both nitrogen and phosphate contents identical between both fresh modified Bristol medium and flocculated medium, extra NaNO_3_ (197.2 mg/L) and KH_2_PO_4_ (217.6 mg/L) were supplemented into the flocculated medium for the re-cultivation of the next batch of microalgal cells. In addition, there is no difference for the nutrient addition between chitosan and aluminum sulfate flocculated medium. Fresh modified Bristol medium was applied to grow microalgae as the control group. The recycled and control media were inoculated with 10% v/v of seed microalgal suspension with the OD_680_ of 3.012. Microalgal growth was monitored and optical density was measured at 2 days interval.

### Lipid extraction

In order to determine effects of the harvesting approaches by using traditional flocculant and natural flocculant on the extraction of lipids, centrifugation was also applied to harvest microalgal biomass. Microalgae cells were collected and centrifuged at 5000 rpm for 15 min. Supernatants were decanted, and cell pellets were washed with distilled water and then dried to achieve a constant weight. The dried microalgal biomass samples after flocculation and centrifugation were collected and sealed in empty containers for lipid extraction analysis.

According Zhu et al. [10], 100–150 mg freeze-dried algal samples were weighed and extracted with 2 mL methanol containing 10% dimethyl sulfoxide (DMSO) in a water bath shaker at 45 °C for 45 min. The mixture was centrifuged at 3000 rpm for 10 min. Then, the supernatant was collected and leftover was re-extracted twice following the same process. Afterwards, the leftover was extracted with 4 mL mixture of hexane and ether (1:1, v/v) in a water bath shaker at 45 °C for 60 min. The mixture was centrifuged at 3000 rpm for 10 min. Then, the supernatant was collected and leftover was re-extracted twice following the same process. All the supernatants were combined, after which 6 mL water was added to the incorporated extracts to form a ratio of methanol with 10% DMSO, diethyl ether, hexane and water of 1:1:1:1 (v/v/v/v). The organic phases with lipids were transferred into a pre-weighed glass tube and evaporated to dryness under nitrogen protection. Subsequently, the lipids were freeze-dried under − 80 °C for 24 h. Afterwards, the total lipids were determined gravimetrically, and lipid content was expressed as % of dry weight.

All the experiments in this study were carried out in duplicate and average values were reported. Results were performed with EXCEL and SPSS 11.5 for Windows.
